# Parameters of Micro- and Macrocirculation in Young Uncomplicated Type 1 Diabetic Patients—The Role of Metabolic Memory

**DOI:** 10.3390/ijms262010156

**Published:** 2025-10-18

**Authors:** Jolanta Neubauer-Geryk, Małgorzata Myśliwiec, Katarzyna Zorena, Leszek Bieniaszewski

**Affiliations:** 1 Clinical Physiology Unit, Medical Simulation Centre, Medical University of Gdańsk, 80-204 Gdańsk, Poland; lbien@gumed.edu.pl; 2 Department of Pediatrics, Diabetology and Endocrinology, Medical University of Gdańsk, 80-210 Gdańsk, Poland; mysliwiec@gumed.edu.pl; 3 Department of Immunobiology and Environment Microbiology, Medical University of Gdańsk, 80-210 Gdańsk, Poland; kzorena@gumed.edu.pl

**Keywords:** metabolic memory, macrocirculation, microcirculation, early microvascular alterations, rarefaction, capillaroscopy, immune profile, type 1 diabetes mellitus, pediatric patients

## Abstract

In the current study, we focus on analyzing the relationship between changes in micro- and macrocirculation and different stages of metabolic memory. We hypothesized that early poor glycemic control induces lasting endothelial changes detectable in pediatric type 1 diabetes (T1D) microcirculation. We assessed microcirculation structure and function using capillaroscopy, transcutaneous oxygen pressure (TcPO_2_), and optical coherence tomography (OCT). We evaluated macrovascular circulation using pulsatility index (PI), ankle-brachial index (ABI) and pulse pressure (PP). We also examined the relationship between circulation parameters, the age at onset, and diabetes duration. The study included 67 patients with uncomplicated type 1. We divided all patients into four groups based on their HbA_1c_ levels at T1D onset and their average HbA_1c_ after one and two years. We assessed the concentrations of TNF-α, IL-35, IL-4, IL-10, IL-18, IL-12, serum angiogenin, VEGF, sVCAM-1, ICAM-1, sP-Selectin, AGEs, and sRAGE. We compared subgroups with different levels of metabolic memory but comparable T1D duration and age at diagnosis. Micro- and macrovascular parameters were similar between the groups. Our comparison of subgroups with identical metabolic memory but different durations and ages at diagnosis revealed clear differences. The subgroup with a shorter T1D duration showed higher capillary density and a smaller inter-capillary distance compared to those with a longer diabetes duration. This subgroup with shorter duration had significantly lower AGE levels and a reduced TNF-α/IL-35 ratio, along with higher levels of IL-35, IL-4, and IL-12, compared to the longer-duration group. Our findings indicate that in youths with uncomplicated T1D, disease duration—not metabolic memory—plays a dominant role in early microvascular alterations.

## 1. Introduction

Type 1 diabetes (T1D) poses a major global health challenge, especially as its prevalence increases among young children [1]. It is well known that the primary cause of long-term complications is the development of biochemical changes caused by hyperglycemia. This damage continues to worsen even after good blood glucose control is achieved. Therefore, the early intensive treatment and strict management of blood glucose are crucial for preventing both macrovascular and microvascular complications.

Metabolic memory is a well-established concept that describes the long-lasting effects of early, sustained high blood glucose levels, which can cause permanent damage to blood vessels [2,3,4,5,6,7].

There is a noticeable lack of research on metabolic memory and vascular complications in children with type 1 diabetes. The Diabetes Control and Complications Trial (DCCT) focused on assessing the risk of classic microangiopathic complications [6]. Additionally, other studies have examined biochemical prognostic factors related to the development of microangiopathy in T1D [8]. Research has shown that endothelial dysfunction, indicated by impaired flow-mediated dilation, is common among adolescents with type 1 diabetes diagnosed for less than five years. This dysfunction is associated with the duration of the disease, the presence of microalbuminuria, and the average HbA_1c_ level during the second year of diabetes. However, it is not linked to the mean HbA_1c_ level during the first year [9]. However, the relationship between early vascular complications and metabolic memory in children and adolescents has yet to be investigated.

Metabolic memory is a well-established concept that describes the long-lasting effects of early, sustained high blood glucose levels, which can cause permanent damage to blood vessels. This phenomenon is characterized by a series of complex biochemical processes initiated by prolonged hyperglycemia, leading to increased glycation of proteins and lipids. The severity and duration of high blood glucose levels are reflected in the accumulation of advanced glycation end products (AGEs), which seem to contribute to endothelial cell damage, trigger inflammatory responses, and induce oxidative stress [10]. These harmful effects can persist long after blood glucose levels return to normal. Endothelial cell dysfunction occurs early in type 1 diabetes as a result of hyperglycemia [11]. This dysfunction then reduces the ability to grow new blood vessels in response to tissue ischemia. Data have shown that poor blood glucose control in people with diabetes is linked to increased oxidative stress and higher levels of protein glycation [12]. Recent studies suggest that cytokines play an important role in disease development [13,14,15]. Additionally, many inflammatory markers under investigation show gradual changes over time. Studies using experimental models of metabolic memory reveal that epigenetic mechanisms influence the negative effects associated with hyperglycemia [4,16,17,18,19].

Diabetes duration and age at onset are key factors in macroangiopathy; their effects are amplified by modifiable risk factors often observed in children with T1D.

The current study builds upon our previous research on metabolic memory, as described in our article published in the International Journal of Medical Sciences [20]. In this report, we showed that both metabolic memory and inflammation contribute to an altered lipid profile in these patients. We found that poor glycemic control at the onset of diabetes and during the first two years of the disease was significantly correlated with an imbalance between pro-inflammatory and anti-inflammatory cytokines. These findings were the basis for formulating the rationale for the current study, in which we focus on analyzing the relationship between changes in micro- and macrocirculation and different stages of metabolic memory.

Our present study is focused on analyzing the impact of metabolic memory on micro- and macrocirculation parameters. Microcirculation structure and function were assessed using capillaroscopy and transcutaneous oxygen pressure (TcPO_2_) [21], and optical coherence tomography (OCT). Macrocirculation was assessed using pulsatility index (PI), as well as the ankle–brachial index (ABI) and pulse pressure (PP). Moreover, we analyzed the effect of age at onset and disease duration on these parameters. We hypothesized that early poor glycemic control induces lasting endothelial changes detectable in pediatric T1D microcirculation.

## 2. Results

**Figure 1:**
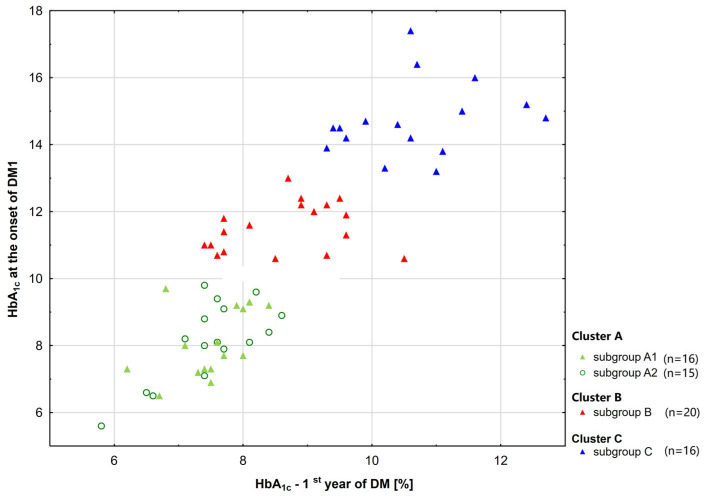
Patient groups based on HbA_1c_ levels at disease onset, and mean HbA_1c_ values obtained after 1st and 2nd year following diagnosis. The triangles and circles indicate median values [20].

Initially, sixty-seven (67) patients with type 1 diabetes of Polish nationality, including 37 girls and 30 boys, participated in the study. Patients were divided into three groups based on their HbA_1c_ levels at diagnosis and their average levels during the first and second years after diagnosis (see Figure 1). Our previous article, “The Impact of Metabolic Memory on Immune Profile in Young Patients with Uncomplicated Type 1 Diabetes,” provided a detailed description of this process [20].

**Figure 2:**
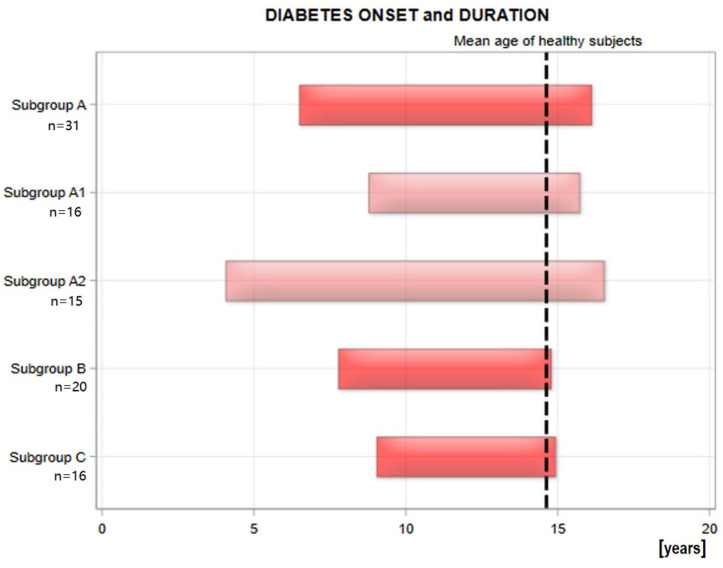
Demographic data in subgroups of diabetic patients in each group [20].

Clusters A, B, and C of patients were similar in terms of age and age of onset (see Figure 2). To create clusters with similar diabetes duration, Cluster A was split into two subgroups: A1 and A2. This was done using the median diabetes duration (9.87 years) as a cutoff point. Demographic characteristics of subgroup A1 are comparable to those of subgroups B and C. Therefore, there are four subgroups: A1, A2, B, and C. First, a comparison was made between subgroups with different metabolic memory but similar diabetes duration and age of onset (A1, B, C). The subgroups did not exhibit significant differences in terms of patient gender. Next, we compared subgroups with the same metabolic memory but different durations and ages of onset of diabetes (A1 and A2).

### 2.1. The Whole Study Group and Subgroups (A1, B, C) [20]

The subgroups (A1, B, C) studied were similar in terms of BMI, age, disease duration, age of onset, and the amount of insulin units administered per kilogram of body weight. The number of mild and severe hypoglycemic episodes, as well as the percentage of pump use in relation to disease duration and the number of autoimmune comorbidities (celiac disease and autoimmune thyroiditis) were not different.

The only differences between the A1 and C groups were in total cholesterol and LDL cholesterol levels. Patients in the A1 group had significantly lower total and LDL cholesterol levels than those in the C group.

The subgroup analysis showed that group A1 had significantly higher IL-35 and IL-10 levels, and lower TNF-α and TNF-α/IL-35 ratio than group C. Patients in group A1 also had higher IL-35, IL-4, and IL-10, and a lower TNF-α/IL-35 ratio than group B. Patients in group B had higher TNF-α levels than group C. The other biomarkers examined showed no differences between groups A1 and B, B and C, or A1 and C. Additionally, group A1 had significantly lower VEGF and AGEs concentrations than groups B and C.

An analysis of the macrocirculation parameters, including pulsatility indices at several locations (carotid, brachial, thigh, above and below the knee, and at the ankle), showed no significant differences among groups A1, B, and C. There were also no differences in ankle–brachial index, systolic, diastolic, and pulse pressure between these groups.

An analysis of the microcirculation parameters, including capillaroscopy, transcutaneous oxygen pressure, and optical coherence tomography, showed no significant differences between groups A1, B, and C.

### 2.2. Comparison of A1 and A2 Subgroups

The selected groups of diabetic patients did not differ significantly in age, HbA_1c_ levels at the onset of T1D, or in the first and second years of the disease (Figure 1). There was also no significant difference in present HbA_1c_ levels. The test power is 94.8.

They did not differ significantly in the duration of insulin pump use, insulin dose per kilogram of body weight, body mass index (BMI), or the number of mild and severe hypoglycemic episodes (Table 1). The study groups differed in terms of age at onset and diabetes duration (Figure 2).

#### 2.2.1. Characteristics of Groups A1 and A2

The patient groups did not differ in BMI, age, HbA_1c_ at diabetes onset, mean HbA_1c_ during the first and second years, current HbA_1c_ levels, or insulin dose units/kg. Groups A1 and A2 were evenly distributed by gender, and treatment with a pump had comparable rates of hypoglycemia, autoimmune thyroiditis, and celiac disease (Table 1).

#### 2.2.2. Laboratory Examination

The groups examined showed no significant differences in lipids and creatinine levels, C-reactive protein (CRP), thyroid hormones, or albumin (Table 2).

#### 2.2.3. Biomarkers Examination

The study indicated that the only difference was in the AGEs level, which was significantly lower in Group A1. IL-35, IL-4, and IL-12 levels were significantly higher, while the TNF-α/IL-35 ratio was significantly lower in group A1. The levels of angiogenin, vascular endothelial growth factor (VEGF), soluble P-selectin (sP-selectin), and soluble receptor for advanced glycation end products (sRAGE) as well as the levels of sVCAM-1 and ICAM-1, or TNF-α and IL-18 (interleukin-18) and IL-10 (interleukin-10) were similar between group A1 and group A2 of patients with type 1 diabetes (Table 3).

#### 2.2.4. Pulsatility Indices and Pulse Pressure

The patient groups showed no difference in the pulsatility index of elastic and muscular arteries. The ABI and pulse pressure also did not differ between the groups studied (Table 4).

#### 2.2.5. Microcirculation

A comparative analysis of the indicators obtained during capillaroscopy, transcutaneous oxygen pressure measurement, and optical coherence tomography showed that the group with a shorter diabetes duration (A1) has more efficient skin microcirculation, reflected by higher capillary coverage and a shorter distance between capillaries (Table 5). This finding suggests that microvascular structural alterations precede functional changes in these patients.

## 3. Discussion

Prolonged hyperglycemia leads to the formation of AGEs, which increase vessel stiffness and impair blood flow, especially in small vessels [22], resulting in tissue hypoxia. Therefore, effective glycemic control is crucial for preventing AGE formation and microcirculatory complications. The damaging mechanisms are more complex than just the effect of AGEs [23].

In our study, patients with different capillary densities exhibit similar levels of the receptor for AGEs (RAGE). This indicates that oxidative stress and mitochondrial dysfunction in endothelial cells also contribute to vascular damage. Genetic predispositions can hasten capillary loss even in individuals with similar metabolic profiles. It has been demonstrated that serum AGEs are linked to the presence of retinopathy, nephropathy, or neuropathy. Serum AGEs also enable the prediction of these microvascular complications [24,25]. Our current study revealed no differences in micro- and macrocirculation parameters between subgroups of patients with different levels of metabolic memory but similar diabetes duration and age at onset (A1, B, C). Patients with the best metabolic memory had significantly lower total and LDL cholesterol levels, better anti-inflammatory cytokine profiles, and lower AGE concentrations.

Capillary density can act as an early sensitive indicator of microcirculatory damage in young diabetic patients. In the early stages of diabetes, capillary density may even increase as a compensatory response. As the condition progresses, it is observed that capillary density declines [26].

Our data indicate that the local inflammatory environment directly affects vascular integrity. It shows that lower AGE levels are linked to preserved capillary density in young, uncomplicated type 1 diabetic patients. Additionally, in patients with higher capillary density (A1), increased levels of anti-inflammatory cytokines (IL-35 and IL-4) and decreased levels of pro-inflammatory factors, such as TNF-α, were observed. This positive biochemical profile was significantly different from that of patients with lower capillary density (A2). It suggests that microcirculation damage, exemplified by capillary loss (coverageBASE), and biochemical changes—like cytokine and AGE levels—may occur before any signs of functional decline are visible. There is also a relationship between the age of onset, duration of diabetes, and differences in microcirculation parameters.

The metabolic and inflammatory markers are similar across patient groups that differ in age at onset and diabetes duration. However, differences in capillary density and cytokine profiles suggest distinct mechanisms underlying early microcirculatory damage. To better understand microangiopathy, it is crucial to analyze the dynamics within the local vascular environment and individual immune responses. Exploring these complex processes will help develop new, more targeted treatments. Our research clearly shows that patients with a long duration of diabetes (9.9–15.9 years) and earlier disease onset (1.2–7.6 years) experience significant deterioration in microcirculation function.

Research by Cisło et al. showed that changes in skin microcirculation in young patients with type 1 diabetes are independent of age at onset but worsen with longer disease duration [27]. Others have also demonstrated a correlation between the duration of diabetes and changes observed in capillaroscopy images [28,29,30,31,32,33]. Tibiriçá and colleagues found that patients with T1D do not exhibit thinning of skin capillaries. Furthermore, this report revealed a lack of capillary reserve [34]. Tooke et al. demonstrated that microcirculation changes in T1D patients are time-dependent [35]. Skin microvascular autoregulatory responses are impaired within the first year of diabetes and become more pronounced after 10 years of disease. In contrast, Trapp et al. [36] found no differences in the morphology, number, or density of capillaries in long-term type 1 diabetes (T1D) compared to controls. Our previous studies also indicated that in adult patients with type 1 diabetes, capillary reactivity was not correlated with the duration of diabetes but was negatively correlated with the age at onset [37].

The retinal microcirculation assessed through OCT revealed no significant differences between patients regarding disease duration or age at onset. Similarly, research conducted by Gołębiewska et al. found no statistically significant correlations between the vessel densities of the superficial capillary plexus (SCP) and deep capillary plexus (DCP) in relation to disease duration or age of onset [38]. In contrast, other studies have reported a negative correlation between the total density of the retinal superficial capillary plexus [39,40] and deep capillary plexus with the type 1 diabetes duration [39].

Diabetes significantly contributes to increased arterial stiffness through multiple physiological mechanisms. The reduced availability of nitric oxide causes endothelial dysfunction and chronic inflammation [41]. Additionally, diabetes affects the composition of connective tissue in arterial walls through processes like collagen cross-linking and increased collagen glycation [12]. These changes are further exacerbated by factors such as high blood pressure, dyslipidemia, and persistent hyperglycemia. Notably, aging has a more significant negative effect on proximal and elastic arteries compared to distal and muscular arteries. Elevated lipid levels are a risk factor for vascular stiffness and aging, affecting arterial properties. Our study shows that macrovascular indicators are similar, regardless of any metabolic memory or substantial differences in total and LDL cholesterol levels.

Both elastic and muscular arteries can be evaluated using the pulse index and ankle–brachial index. The pulsatility index also assesses microcirculation function. It is a quick, simple, and non-invasive test that requires minimal cooperation, making it suitable for use in children. The relationship between age at onset and disease duration is complex. Early onset not only increases the risk of severe disease but also prolongs its course, leading to more exposure to cardiovascular risks. This accumulated exposure during adolescence is important for early vascular damage, which is often more severe in individuals with poor glycemic control or additional risk factors such as dyslipidemia or obesity.

Early-onset type 1 diabetes (T1D) significantly affects long-term health and increases the risk of developing cardiovascular disease by thirtyfold due to prolonged exposure to various risk factors. Patients diagnosed with T1D before the age of 10 may lose an average of 17.7 years of life for women and 14.2 years for men compared to healthy individuals [42]. Prolonged exposure to risk factors and possibly faster disease progression likely explain the higher complication rates observed in early-onset cases [43].

The early onset of the disease and a longer duration are essential factors in developing early vascular changes in pediatric patients with type 1 diabetes. Disease duration independently predicts arterial stiffness (PWV) [44], while the age at onset is associated with increased intima-media thickness (IMT), impacting various aspects of early vascular damage [45]. Although clinical signs of cardiovascular disease (CVD) are rare during childhood, the underlying processes begin to develop earlier, during childhood and adolescence. This early onset accelerates the development of atherosclerosis, increasing the risk for those with T1D [46]. Our analyses showed no differences in parameters measuring arterial stiffness among the three groups with different levels of metabolic memory, nor between groups with similar metabolic memory but different ages at diabetes onset and disease durations. This likely results from the relatively short diabetes duration. It can also suggest that dysfunction in microcirculation precedes structural and functional changes in macrocirculation. Additionally, the young age of the patients (children) may indicate that their large blood vessels are still relatively elastic, enabling them to compensate for early damage effectively. Concerning the ankle–brachial index (ABI), it appears to reflect later vascular changes.

The cross–sectional nature of our study limits the possibility of concluding cause and effect. The absence of microangiopathic complications in children and adolescents with diabetes in this study highlights both its limitations and benefits. Although a broader range of biomarkers could have been examined, the study’s scope was limited by planning and financial constraints. Additionally, we did not assess IMT, which could have provided valuable insights into changes in the large vessel structure. It is essential to conduct follow-up assessments to verify the presence of early microvascular markers.

## 4. Materials and Methods

### 4.1. Study Design and Population

The study included patients with diabetes who had lasted at least 1.2 years. Patients were recruited from the Department of Pediatrics, Diabetology, and Endocrinology of the Medical University of Gdańsk between 2014 and 2018.

Patients met the diagnostic criteria for type 1 diabetes, as defined by the International Society for Pediatric and Adolescent Diabetes. For all diabetic patients, the average age at diagnosis was 8.5, with an average duration of diabetes of 6.6 years. The exclusion criteria for the study included micro- and macrovascular complications, acute diabetes issues, abnormal TSH and free thyroxine levels, systemic diseases such as rheumatoid arthritis and psoriasis, and the use of statins.

Severe hypoglycemia was defined as an episode of blood glucose below 54 mg/dL that required assistance from another person. This occurred within the year and no later than one month before the exam. Mild hypoglycemia was defined as episodes of blood glucose below 54 mg/dL during the last month and did not require intervention [47].

An experienced pediatrician conducted a physical exam. We used consistent standards to confirm the absence of microangiopathy. Diabetic nephropathy was diagnosed based on twice-documented albuminuria over 30 mg/day in the past 6 months [47,48].

After a detailed explanation of the study’s purpose and procedures, the patients provided informed consent. Parents agreed to their child’s participation and were present during the examinations.

The research methodology was approved by the Independent Bioethics Committee for Scientific Research at the Medical University of Gdańsk (decisions NKBBN/277/2014 of 8 July 2014, and NKBBN/277-512/2016 of 5 December 2016).

### 4.2. Laboratory Analysis

Laboratory tests were performed as previously described [49]. Detailed descriptions are provided here for the readers’ convenience.

Blood samples were collected from study participants between 7:00 and 9:00 a.m. after an overnight fast. Venous blood was drawn, and the serum was separated and stored frozen at −80 °C for up to three months before analysis. The data presented here were obtained from a single sample.

The HbA_1c_ level was measured using immunoturbidimetry (Hoffmann-La Roche AG, Basel, Switzerland). The fasting blood glucose level was determined through an enzymatic test (Roche Diagnostics GmbH, Mannheim, Germany). The C-reactive protein level was assessed using an immunochemical system (Beckman Instruments, Inc., Galway, Ireland). Total cholesterol, HDL and LDL cholesterol, and triglyceride levels were measured with Cormay enzyme kits (Cormay, Lublin, Poland). Serum creatinine levels were determined using the CREA system (Boehringer Mannheim GmbH, Mannheim, Germany). All tests were performed at the certified University Laboratory Centre in Gdańsk. The serum levels of IL-4, IL-10, and IL-18, TNF–α, and IL-12, VEGF A, ICAM-1, sVCAM–1, sP-selectin, angiogenin level, and sRAGE concentrations were measured using an ELISA kit (Quantikine High Sensitivity Human from R&D Systems, Minneapolis, MN, USA) according to the manufacturer’s protocol. The quantification of human IL-35 was performed using an ELISA assay (Thermo Fisher Scientific, Inc., Waltham, MA, USA). Serum AGEs concentrations were measured using an ELISA test (USCN Life Science Inc., Wuhan, China) according to the manufacturer’s protocol.

### 4.3. Pulsatility and Ankle–Brachial Indices, and Pulse Pressure

Blood pressure measurements were conducted in accordance with the guidelines established by the European Federation of Internal Medicine [50]. The calculation of pulse pressure was determined by the difference between systolic and diastolic blood pressure values. An arterial pulsatility test was administered as previously outlined in the literature [21,49]. For the benefit of the readers, a detailed description of the methodology is provided here. The test was performed in a controlled environment maintained at an approximate temperature of 20 °C. Before the 30 min evaluation, participants were instructed to rest in a supine position for 10 min.

Flow characteristics were assessed in the common carotid artery, brachial artery, and the arteries of both the upper and lower limbs using the VasoGuard 5000 device (Ni-coletes, Image Monitoring Inc., Mississauga, ON, Canada). A cuff was strategically placed on the lower leg at four specific locations: upper thigh, above the knee, below the knee, and above the ankle. Measurements were taken simultaneously on both sides of the body. The VasoGuard 5000 software automatically calculated the pulse index for each artery examined, from which the average index value for both sides was derived. Additionally, the common carotid artery was measured three times on each side [21,49]. All VasoGuard exams were performed by the same physician. The ankle–brachial index was determined through automated calculations facilitated by the VasoGuard 5000 [21].

### 4.4. Capillaroscopy

The capillary examination procedure is thoroughly documented to ensure accuracy and reliability [34,37]. Participants were advised to avoid manicures two weeks before the exam. The assessment was conducted while participants sat comfortably, with their arms supported and hands placed under a capillaroscope. To ensure precise measurements, body temperature was monitored using a non-contact thermometer (model: Novama-NT), with all participants maintaining normal temperature ranges. The room temperature was kept at a constant level through air conditioning. Images were captured with a digital camera (5 MP; OPTA-TECH, Warsaw, Poland) connected to a capillaroscope (OPTATECH, CS-CREATIVE SOLUTIONS Group, Warsaw, Poland) and stored securely. These images were analyzed using specialized software to calculate the ratio of visible capillary area to the total image area, referred to as coverage. The distance between capillaries within the analyzed area was also measured before and after the PORH test [21].

**Figure 3:**
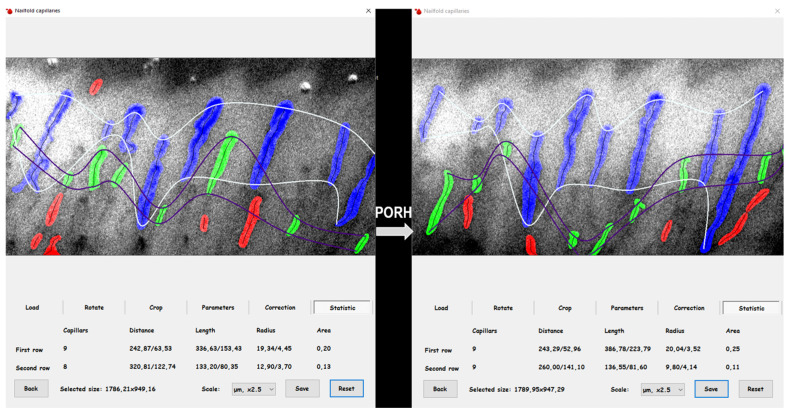
The result of the analysis of capillaroscopy images after PORH test (“,” the comma used in numbers corresponds to the decimal separator; description in the text).

Skin microcirculation reactivity was assessed using a post-occlusive reactive hyperemia (PORH) test after a 20 min resting period (Figure 3). A blood pressure cuff was placed on the non-dominant arm and inflated to a pressure of 50 mmHg above the systolic blood pressure, occluding blood flow for four minutes [51]. Capillary images were recorded before the test (coverage_BASE_) and immediately after the PORH test (coverage_PORH_). The differential in capillary coverage area (∆coverage) was utilized to assess skin microcirculation reactivity before and after the PORH test (∆coverage_PB_). This methodical approach enhances the understanding of skin microcirculation.

### 4.5. Transcutaneous Oxygen Pressure

The patient’s forearm was used to measure transcutaneous oxygen pressure in the same position and environment described for capillaroscopy. The sensor was placed in the middle of the forearm after it was cleaned. The PeriFlux 5000 device (Perimed AB, Järfälla, Sweden) measured transcutaneous oxygen pressure. Transcutaneous partial oxygen pressure (TcPO_2_) is based on the amount of oxygen that diffuses from the capillaries through the epidermis to the electrode. This measurement is a definitive indicator of the body’s capacity to deliver oxygen to its tissues. The methodology for measuring TcPO_2_ has been previously discussed. Measurements are performed following a minimum resting period of 20 min, during the application of occlusion, and after cuff deflation [21]. The curve analysis identified specific points, including TcPO_2__base (the start of occlusion), TcPO_2__zero (the end of occlusion), and TTR (the time to reach baseline after occlusion), which indicate the moment of return to the initial state. The patient’s forearm was selected as the site for measuring transcutaneous oxygen pressure, following the positioning and environmental conditions described for capillaroscopy. The sensor was placed in the middle of the forearm after confirming the area was clean and hair-free. The PeriFlux 5000 device (Perimed AB, Järfälla, Sweden) was used to measure TcPO_2_.

### 4.6. Optical Coherence Tomography

The evaluation of early retinal changes was performed using optical coherence tomography (OCT) with equipment from Topcon, Tokyo, Japan [52]. Mean cube thickness (TAC) and central subfield thickness (CST) were measured following the Early Treatment Diabetic Retinopathy Study protocol. TAC represents the average thickness of the retina within a defined, cube-shaped area of the three-dimensional scan. The volume of the cube reflects the total retinal volume within that region. This method allows for the calculation of retinal tissue volume by multiplying the thickness at each measurement point by the area covered by the scan.

CST represents the average thickness of the retina within the central macular zone, which is defined by a 1 mm diameter circle centered on the fovea. TAC and CST values were determined by averaging data from both the left and right eyes [53].

### 4.7. Statistical Analysis

All statistical analyses of the data obtained were performed using SAS^®^ OnDemand for Academics, SAS Institute Inc., SAS Campus Drive, Cary, NC, USA. The clusters were identified using the FASTCLUS procedure in SAS. The distribution of variables was assessed with the Shapiro–Wilk test. For normally distributed variables, indicated by their mean and standard deviation (SD), Student’s *t*-test was used. The Mann–Whitney U test was used for variables that did not follow a normal distribution. The relationships between variables were analyzed with two statistical methods: Spearman’s rank correlation and the Chi-square test. The Chi-square test was used to compare the distribution and frequency of hypoglycemic episodes. The analysis included all data points, without excluding outliers, and the significance level was set at *p* < 0.05.

## 5. Conclusions

Our findings indicate that in youths with uncomplicated T1D, disease duration—not metabolic memory—plays a dominant role in early microvascular alterations. The protection of microcirculation is essential. In young patients, early therapeutic interventions should emphasize not only strict glycemic control but also the preservation of microcirculation from structural changes. Therefore, an early assessment of microcirculation using non-invasive methods, such as capillaroscopy, is crucial for identifying at-risk patients before complications manifest clinically.

It is recommended that capillaroscopy be integrated into the standard of care for Type 1 Diabetes Mellitus, analogous to its established role in rheumatology, to enable the detection of complications at a stage earlier than classic microangiopathy.

## Figures and Tables

**Table 1:** Characteristics and comparisons of the study groups [20].

Characteristics	Diabetic Patients		*p*
	Group A1 n = 16	Group A2 n = 15	
Males, n (%)	4 (27)	9 (56)	0.17
BMI [kg/m^2^]	20.4 (15.3–26.0)	22.6 (16.1–25.6)	0.30
Age [years]	17.1 (8.4–18.0)/15.7 ± 2.9	17.1 (13–17.9)/16.5 ± 1.6	0.65
Onset of diabetes [age]	9.1 (3.1–13.5)/9 ± 3.2	3.8 (1.2–7.6)/4.2 ± 2	***
Diabetes duration [years]	7 (1.2–9.4)/6.7 ± 2.4	12.2 (9.9–15.9)/12.3 ± 1.8	***
Insulin dose units/24 h	40 (21–70)	54 (30–100)	*
Insulin dose units/kg	0.7 (0.4–1)	0.8 (0.6–1.4)	0.10
Treatment with pump [%]	80 (0–90)	76 (0–100)	0.45
HbA_1c_ at onset of T1D [%]	7.7 (6.5–9.7)	8.15 (5.6–9.8)	0.63
Mean HbA_1c_ 1st year of T1D [%]	7.5 (6.2–8.4)	7.5 (5.8–8.6)	0.98
Mean HbA_1c_ 2nd year of T1D [%]	7.8 (5.5–10.1)	7.0 (6.2–8.6)	0.30
HbA_1c_ current [%]	6.8 (5.9–11.6)	8.6 (6.2–9.8)	0.07
Episodes of mild hypoglycemia [N/last month]	10 (0–16)	10 (0–20)	0.83
Episodes of severe hypoglycemia [N/last year]	0 (0–1)	0 (0–1)	0.74
Autoimmune thyroiditis, n [%]	5 (31)	4 (27)	0.90
Celiac disease, n [%]	4 (25)	4 (27)	0.76

Data are presented as median (range)/mean values ± SD. The value of *p* < 0.05 was regarded as statistically significant. * (*p* < 0.05), *** (*p* < 0.001). Abbreviations: HbA_1c_—glycated hemoglobin; BMI—body mass index; T1D—diabetes mellitus.

**Table 2:** Characteristics and comparisons of laboratory examination in the study groups [20].

Characteristics	Diabetic Patients		*p*
	Group A1 n = 16	Group A2 n = 15	
CRP [mg/L]	0.6 (0.2–4.8)	0.3 (0.1–1.3)	0.09
Serum creatinine [mg/dL]	0.71 (0.45–0.86)	0.77 (0.5–0.95)	0.57
Albuminuria [mg/dL]	14 (3–33)	7.1 (2.5–88)	0.28
Total cholesterol [mg/dL]	164 (129–248)	182 (127–247)	0.25
Cholesterol LDL [mg/dL]	95 (61–138)	106 (61–170)	0.30
Cholesterol HDL [mg/dL]	52 (33–63)	53 (39–75)	0.77
Triglycerides [mg/dL]	66 (34–294)	82 (45–159)	0.32
TSH [mIU/L]	1.6 (0.6–3.9)	2 (1–4.3)	0.57
fT4 [pmol/L]	12.8 (11.3–15)	12.9 (10.4–15.0)	0.83

The value of *p* < 0.05 was regarded as statistically significant. Abbreviations: CRP—C-reactive protein; LDL—low-density lipoproteins; HDL—high-density lipoproteins; TSH—thyroid-stimulating hormone; fT4—free thyroxine.

**Table 3:** Biomarkers in the study groups. Data are shown as the median with the range.

Characteristics	Diabetic Patients		*p*
	Group A1 n = 16	Group A2 n = 15	
anti-inflammatory cytokines [20]			
IL-35 [ng/mL]	11.9 (3.9–22.7) 11.95 #	4.7 (0.8–11.4) 3.81 #	***
IL-4 [pg/mL]	14.2 (2.2–29)/ 8.4 #	4.2 (0.7–17.8) 6.5 #	**
IL-10 [pg/mL]	1.8 (0.5–4.7)	1.6 (0–3.8)	0.47
pro-inflammatory cytokines [20]			
TNF–α [pg/mL]	1.6 (0–3.5)	1.9 (0–4.9)	0.63
IL-12 [pg/mL]	4.4 (0–16.3) 5.3 #	3.9 (0–9.2) 7.6 #	*
IL-18 [pg/mL]	78.2 (34.6–146)	77.2 (45–124.6)	0.87
ratio TNF–α/IL-35	0.09 (0–0.43) 0.15 #	0.4 (0–2.97) 0.6 #	***
Vascular and inflammatory biomarkers			
Serum angiogenin [ng/mL]	298.3 (124–799.6)	257.6 (103–985)	0.50
VEGF [pg/mL]	132 (55.6–6848)	180.6 (75.5–363.8)	0.23
sVCAM–1 [ng/mL]	301 (77–337.9)	326.5 (98.5–654)	0.71
ICAM–1 [ng/mL]	263.6 (115.6–389.4)	228.9 (177.5–471.9)	0.68
sP-Selectin [ng/mL]	298.3 (98.4–521.6)	260.9 (146–731.6)	0.68
AGEs [pg/mL]	13,450 (5,48–24,560) 9174 #	22,033 (7650–32,520) 11,595 #	**
sRAGE [pg/mL]	1287 (788–2483)	1304 (1106–1982)	0.50

The value of *p* < 0.05 was regarded as statistically significant. * (*p* < 0.05), ** (*p* < 0.01), *** (*p* < 0.001), #—interquartile range. Abbreviations: TNF–α—tumor necrosis factor; IL-35—interleukin 35; IL-4—interleukin 4; IL-10—interleukin 10; IL-12—interleukin 12; IL-18—interleukin 18; ratio TNF–α/IL-35—the ratio of TNF–α and IL-35; VEGF—vascular endothelial growth factor; sVCAM–1—Soluble Vascular Cell Adhesion Molecule–1; ICAM–1—Intercellular Adhesion Molecule–1; sP-Selectin—Soluble Platelet Selectin; AGEs—Advanced Glycation End Products; sRAGE—Receptors for Advanced Glycation End Products.

**Table 4:** Pulsatility indices and pulse pressure in the studied groups. Data are shown as the median with the range.

Characteristics	Diabetic Patients		*p*
	Group A1 n = 16	Group A2 n = 15	
CCA_PI	2 (1.4–2.7)	1.8 (1.4–2.9)	0.49
brachial_PI	2.5 (1.8–3)	2.4 (1.8–3.7)	0.83
thigh_PI	2.3 (1.9–2.9)	2.3 (2.0–2.9)	0.77
above_knee_PI	2.3 (2.0–3.2)	2.4 (1.8–3.2)	0.42
below_knee_PI	2.5 (2.1–3.6)	2.7 (2.0–3.6)	0.19
ankle_PI	2.6 (2–3.4)	2.7 (2.2–3.8)	0.42
ABI	1.1 (0.9–1.4)	1.1 (0.9–1.3)	0.68
SBP [mmHg]	107 (89–132)	113 (84–124)	0.19
DBP [mmHg]	58 (49–71)	61 (50–76)	0.28
PP [mmHg]	47 (32–61)	51 (34–70)	0.49

The value of *p* < 0.05 was regarded as statistically significant. Abbreviations: CCA_PI—pulsatility index for common carotid arteries; brachial_PI—pulsatility index for brachial arteries; thigh_PI—pulsatility index for femoral arteries; above_knee_PI—pulsatility index for arteries above knee; below_knee_PI—pulsatility index for arteries below knee; ankle_PI—pulsatility index for arteries at the ankle level; ABI—ankle–brachial index; SBP—systolic blood pressure; DBP—diastolic blood pressure; PP—pulse pressure.

**Table 5:** Capillaroscopy, transcutaneous oxygen pressure, and optical coherence tomography parameters in the studied groups. Data are shown as the median with the range.

Characteristics	Diabetic Patients		*p*
	Group A1 n = 16	Group A2 n = 15	
Capillaroscopy			
Coverage_BASE_ [%]	18 (15.3–21) 3.0 #	15 (12.9–25) 3.1 #	*
Coverage_PORH_ [%]	17 (14.8–21)	16 (10.3–24)	0.57
∆Coverage_PB_ [%]	−1 (−3.1–3)	1 (−8.1–5)	0.28
Capillary reactivity	−5 (−16.4–18)	5 (−41–31)	0.38
Distance_BASE_ [µm]	214.4 (194.5–263.8) 25.4 #	246.5 (179.4–274.9) 31.3 #	*
Distance_PORH_ [µm]	217.6 (195.4–274.5)	241.2 (190.2–345)	0.08
∆Distance_PB_ [µm]	2.8 (−32.8–51.7)	9.7 (−51.5–105.8)	0.86
Transcutaneous oxygen pressure			
TcPO_2__base [mmHg]	58.9 (37.8–77.9)	51.2 (28–71.5)	0.18
TcPO_2__zero [mmHg]	3.3 (1.9–9)	3.4 (1.1–14)	0.54
TTR [s]	90.5 (53–240)	63 (28–240)	0.08
Optical coherence tomography indices			
CST	247 (208–269)	264 (226–285)	0.12
Volume cube	10 (10–11)	11 (10–11)	0.06
TAC	279 (268–310)	292 (273–316)	0.05

The value of (*p* < 0.05) * was regarded as statistically significant, #—interquartile range. Abbreviations: PORH—post-reactive hyperemia; Coverage_BASE_—ratio of the capillary area to the total area of the determined rows in baseline condition; Coverage_PORH_—ratio of the capillary area to the total area of the determined rows after PORH; ∆Coverage_PB_ = Coverage_PORH_–Coverage_BASE_; Capillary reactivity = ∆Coverage_PB_/Coverage_BASE_; Distance_BASE_—mean distance between successive capillaries in baseline condition; Distance_PORH_—average distance between successive capillaries after PORH; ∆Distance_PB_ = Distance_PORH_–Distance_BASE_; TcPO_2__base—mean value of TcPO_2_ within 60 s before T_base; TcPO_2__zero—mean value of TcPO_2_ within 60 s before T_zero; TTR—time to reach the baseline value after occlusion; CST—central subfield thickness; TAC—Thickness Average Cube.

## Data Availability

The data presented in this study are available on request from the corresponding author.

## Abbreviations

HbA_1c_	glycated hemoglobin
VEGF	vascular endothelial growth factor
BMI	body mass index
T1D	diabetes mellitus
TSH	thyroid-stimulating hormone
fT4	free thyroxine
TNF-α	tumor necrosis factor
TGF-β	transforming growth factor beta
ratio TNF–α/IL-35	the ratio of TNF–α and IL-35
IL-	interleukin
ICAM-1	Intercellular Adhesion Molecule–1
sVCAM–1	Soluble Vascular Cell Adhesion Molecule–1
sP–selectin	Soluble Platelet Selectin
AGEs	Advanced Glycation End Products
sRAGE	Receptors for Advanced Glycation End Products
CRP	C-reactive protein
LDL-cholesterol	low-density cholesterol
HDL-cholesterol	high-density cholesterol
IMT	intima-media thickness
CCA_PI	pulsatility index for carotid arteries
brachial_PI	pulsatility index for brachial arteries
thigh_PI	pulsatility index for femoral arteries
above_knee_PI	pulsatility index for muscular arteries above the knee
below_knee_PI	pulsatility index for muscular arteries below the knee
ankle_PI	pulsatility index for muscular arteries at the ankle level
ABI	Ankle–brachial index
SBP	Systolic blood pressure
DBP	Diastolic blood pressure
PP	pulse pressure
PORH	post-reactive hyperemia
Coverage_BASE_	ratio of the capillary area to the total area of the determined rows in the baseline condition
Coverage_PORH_	ratio of the capillary area to the total area of the determined rows after PORH
∆Coverage_PB_	Coverage_PORH_–Coverage_BASE_
Capillary reactivity	∆Coverage_PB_/Coverage_BASE_
Distance_BASE_	mean distance between successive capillaries in baseline condition
Distance_PORH_	average distance between successive capillaries after PORH
∆Distance_PB_	DistanceP_ORH_–Distance_BASE_
TcPO_2__base	mean value of TcPO_2_ within 60 s before T_base
TcPO_2__zero	mean value of TcPO_2_ within 60 s before T_zero
TTR	time to reach the baseline value after occlusion
